# Report Made in the Name of the Committee on Mineral Waters for 1837

**Published:** 1839-06-22

**Authors:** Ph. Patissier


### MEDICAL EXAMINER.

DEVOTED TO MEDICINE, SURGERY, AND THE COLLATERAL SCIENCES.

No. 25.] PHILADELPHIA, SATURDAY, JUNE 22, 1839. [Vol. II.

*Report made in the name of the Committee on Mineral Waters for* 1837. By M. Ph. Patissier.

(Continued from page 376.)

Chap. III.—*On the Chronic Diseases which are oftenest met with at Watering Places.*

We divide chronic diseases, 1st, into those which attack the organs of the head, the chest, and the abdomen; 2d, into those which may be developed in all parts of the body; and, 3d, into surgical diseases. Although this classification is not beyond the reach of a just criticism, we have adopted it because we think it the best adapted to our subject.

Section 1. *Of Chronic Diseases of the Head.*—Mineral waters are rarely used in chronic affections of the brain; it is in this organ that the excitement produced by mineral waters may become dangerous, if the limits are passed within which it ought to be kept to produce favourable effects. Nevertheless, the springs of Bourbonne, of Balaruc, and of Bourbon-l'Archambault, have been for ages past recommended, particularly for paralyses succeeding apoplexy; but they should only be had recourse to when there exists no active congestion towards the brain, and when the patient is rather of a lymphatic than a sanguine temperament. The water ought then to be administered in half-baths of the temperature of the air, in douches upon the paralyzed limbs, and internally, in moderate doses. This treatment, it is true, diminishes the paralysis but slowly, but it prevents the congestions of blood towards the head, which formerly took place frequently at Balaruc, where paralytic patients were plunged for eight successive days in a hot bath.*

When the paralysis is consecutive on a lesion of the spinal marrow, the mineral treatment may be prescribed with more safety and success, without doubt because the circulation of the spinal apparatus is more difficult than that of the brain, and because the organization of the spinal marrow supports better the employment of stimulants. We run a risk, however, of aggravating the state of the patient, if, in paraplegias consequent upon blows or falls upon the posterior part of the trunk, we give douches along the spine; for the percussion of the liquid may reproduce inflammation of the spinal marrow.

STATISTICAL TABLE.

| NAMES OF DISEASES. | *Name of establishment.* | *Number of each disease.* | *Number of diseases cured.* | *Number of diseases relieved.* | *Number of diseases treated without success.* | *Number of diseases cured or relieved after leaving watering place.* |
|---|---|---|---|---|---|---|
| Cerebral hemiplegias, { Right side, 40. Left side, 30. | Balaruc. | 73 | 5 | 40 | 28 | 7 |
| Paraplegias myelites. | Id. | 19 | 2 | 7 | 10 | 2 |
| Cerebral hemiplegias. | Bourbonne. | 41 | 4 | 25 | 12 | 0 |
| Paraplegias. | Id. | 35 | 5 | 27 | 3 | 0 |
| Cerebral hemiplegias. | Bourbon-l'Archambault. | 290† | 20 | 235 | 35 | 0 |
| Paraplegias. | Id. | 210‡ | 68 | 112 | 30 | 0 |
| Cerebral hemiplegias. | Rennes, (Aude.) | 33 | 0 | 3 | 30 | 0 |

M. Faye, the inspecting physician of the springs of Bourbon-l'Archambault, speaks in high terms of the good effects of the cold waters of the chalybeate spring of Jonas, when used in lotions, and especially in douches for incomplete paralysis of the optic nerve. He administers the douche by means of a funnel fixed at a greater or less height in a plank pierced to receive it, the small orifice of which being filled with a sponge, imbibes the water placed above it, and lets it fall, drop by drop, during from five to twenty minutes, upon the middle of the closed eye; this produces a slight excitement, and forces the pupil to contract. M. Faye assures us, that out of two hundred and fifty patients with incomplete amaurosis, whom he attended from 1824 to 1833, forty-four were cured, one hundred and fifty-nine partially relieved, and forty-seven treated without success.

*Neuralgias.*—Douches upon the head, alternately warm and cold, have succeeded several times with M. de Montluc, physician at Neris, for neuralgias of the head, tic doloreux, and rheumatism of the scalp. It is in the treatment of these complaints, that the inspecting physician of Dieppe takes so much credit to himself for the use of sea-baths associated with cold affusions.

* Perhaps these accidents ought to be attributed as much to the heat of the climate as to the heat of the baths.

† From 1824 to 1833. ‡ Id.

*Nervous affections.*—No one is ignorant how seldom it is that nervous affections, such as hypochondria, hysteria, catalepsy, chorea, headach, those of the stomach and intestines, &c., yield to pharmaceutical agents; mineral waters are often the only efficacious remedy, the only consolation which remains for these diseases: they unite all the conditions favourable for their cure, that is to say, change of air, of habits, and of the manner of living, removal from business, and from all causes of care, and, finally, the charms of a new society. The mild and moderately warm waters of Saint-Sauvern, of Ussat, of Salut at Bagnerres de Bigorre, of Bains, of Neris, &c., are useful auxiliaries in treatment both of a moral and hygienic nature; they should be used more externally than internally, and the baths should be tepid, or but slightly cold, and prolonged. The calming power of these waters is so generally appreciated, that the great majority of persons who go to these places is composed of women, in whom, as we know, most diseases are developed under the influence of the nervous system. Those patients who have sufficient strength to enable them to react against the shock of the cold water, find sea-baths, of short duration, beneficial; but it must not be forgotten, in the treatment of nervous affections, that they are rarely idiopathic, and that it is necessary always to seek out the causes of them. Thus, for example, if the nervous symptoms follow upon the suppression or irregularity of a menstrual or hæmorrhoidal flux, or upon the repercussion of a habitual perspiration, or of an exanthema, resort must be had to warm baths, to douches, and to steam baths. When nervous diseases have lasted for some time, they are almost always accompanied (especially hypochondria) with disorder of the lower abdominal viscera; for this complication, the waters of Plombieres, the acidulated waters, and the waters of Vichy, should be prescribed.

STATISTICAL TABLE.

| NAMES OF DISEASES. | *Name of establishment.* | *Number of each disease.* | *Number cured.* | *Number relieved.* | *Number treated without success.* | *Number cured or relieved after leaving the watering place* |
|---|---|---|---|---|---|---|
| Sciatica. | Neris. | 11 | 2 | 5 | 4 | 6 |
| Neuralgia proper. | Id. | 23 | 2 | 17 | 4 | 6 |
| Tremours. | Id. | 1 | 0 | 0 | 1 | 0 |
| Hypochondria. | Id. | 4 | 2 | 2 | 0 | 1 |
| Catalepsy. | Id. | 1 | 0 | 1 | 0 | 0 |
| Neuralgias of different kinds. | Bains. | 14 | 6 | 4 | 4 | 0 |
| Undefined nervous affections. | Id. | 5 | 0 | 3 | 2 | 1 |
| Sciatica. | Mont d'Or. | 7 | 4 | 0 | 3 | 0 |
| Hemicrania. | Id. | 3 | 1 | 2 | 0 | 0 |
| Trifacial neuralgia. | Id. | 4 | 1 | 0 | 3 | 0 |
| St. Vitus' dance. | Id. | 2 | 0 | 1 | 1 | 0 |
| Neuralgia of genital organs. | Id. | 8 | 2 | 4 | 2 | 0 |
| Sciatica, (tic doloreux.) | Bagnères-Luchon. | 45 | 17 | 13 | 15 | 2 |
| Facial neuralgia. | Rennes, (Aude.) | 20 | 2 | 6 | 12 | 4 |
| St. Vitus' dance. | Balaruc. | 6 | 0 | 2 | 4 | 0 |
| Sciatica. | Greoulx. | 17 | 0 | 11 | 6 | 5 |

Section 2. *On Chronic Diseases of the Chest.*—Some mineral springs, such as those of Mont d'Or, of Raillère at Cauterets, of Bonnes, of Manjolet at Arles, of Labassere at Bagnères de Bigorre, have borne a high reputation for the cure of old pulmonary catarrhs, pneumonia, pleurisy in the chronic stage, passive hæmoptysis, of pulmonary phthisis, and commencing laryngitis, as well as of humid asthma; but the rumour of the cures which they have effected often attract to them patients whose cases they do not suit. These waters are salutary in the affections above mentioned, only when they are unaccompanied by dry cough, heat and dryness of skin, and small and frequent pulse, and when there does not exist a too great nervous sensibility; in a word, when the signs of feebleness and relaxation of the animal fibre, prevail over those of irritation. They act, then, by producing a revulsion to the skin, by bringing back the cutaneous secretion to its normal state, by directing the fluids from the centre to the circumference, and by facilitating the expectoration. The relief and cure of diseases are the more certain as the development of the disease is dependent upon the retrocession of some morbid principle, and according as a crisis becomes manifest during or after the treatment in the sweat and stools, as the suppressed flux re-establishes itself, and as exanthemata and boils appear upon the skin. This remark is one of considerable importance, inasmuch as it is necessary in all grave and old affections of the lungs to suspend, or give up entirely, the mineral treatment, if a sudden and notable amendment takes place without the previous occurrence of any critical phenomena; "for," says M. Bertrand, "this momentary and treacherous calm is constantly followed by a redoubled intensity in the pulmonary affection." It is only in phthisis in the first degree, and in a predisposition to this malady, that mineral waters are really useful; they are in these cases much to be preferred to soothing drinks, pectoral syrups, and milk diet.* But if the hæmoptysis is acute, if there is fever, emaciation, and diarrhœa, and if auscultation discovers cavities in the lungs, mineral waters hasten the death of the patient.

The employment of mineral waters is likewise injurious when asthma co-exists with an organic alteration of the heart and large arteries. It is beneficial, on the contrary, in humid asthma succeeding to chronic pulmonary catarrh, or to the metastasis of a rheumatismal or gouty affection. Many asthmatic persons come annually to Mont d'Or, and some are cured, and the greater number notably relieved. There are very few whose attacks do not cease a few minutes after having entered the vapour baths of the thermal establishment. It is in the treatment of this affection that M. Bertrand insists so much upon the patient's passing several hours, whenever the weather permits, in the fir forests hard by the springs of Mont d'Or.

The temperature of the air is also a powerful adjuvant to the medicinal virtues of these waters. In the year 1836, the mean temperature during the months of July and August having been 17° cent., ($62\frac{3}{5}$ Fah.,) M. Bertrand saw several persons with complete aphony cured before their departure from the watering place; the same observation was made with regard to pulmonary, uterine, and auricular catarrhs, chronic rheumatisms, &c.

In the actual state of our knowledge concerning mineral waters, it is difficult to determine in what cases of chronic pulmonary diseases sulphur springs ought to be preferred to those of Mont d'Or. Experience has only taught us that none of these waters ought to be prescribed whenever there exists a too great activity in the functions of the vascular and nervous systems. The waters of Ems, (duchy of Nassau,) which are mild and little exciting, are in such cases better adapted to the state of the patient.

* The waters of Canterets, Bonnes, Bagneres de Luchon, and those of Mont d'Or, are also salutary in cases of *horses* labouring under diseases of the chest. Five horses which coughed violently, were very much emaciated, had the thumps, and were put out of breath by walking a short distance, were brought in 1836 and 1837 to Mount d'Or; they drank from fifteen to twenty litres (4 to 5.2 gallons) of the water every morning for a month; four got completely well, but the fifth died.

We have already said that on account of their exciting effects, none of the mineral waters should be used in the treatment of organic lesions of the circulatory apparatus. We know how pernicious the waters of Pyrénées were to General Foy, when attacked with aneurism of the heart. The palpitations which almost constantly accompany chlorosis, are dissipated by the use of mineral waters, particularly of chalybeates. Those which are the result of a rheumatismal retrocession upon the heart, yield sometimes to baths and douches,—but this treatment requires much prudence and discernment.

Your committee regrets not having received the reports upon the waters of Bonnes and Canterets—it being desirable to compare their therapeutical results with those of the waters of Mont d'Or. We will here remark, however, that it is erroneously, though according with the opinion of Borden, that the waters of Bonnes are considered as being mild and little exciting, so that from three to four pints may be drank during the day, either before breakfast, or before dining, and after this meal. It is certain that, drank in such quantities, they would be promptly fatal to more than three-fourths of the patients who came to be cured by them; that is to say, to those who are affected with chronic diseases of the chest. In these diseases the modifications impressed upon the respiratory organs by the mineral water is the more advantageous as it is developed gradually, and as the stimulation is maintained within just limits. Only a half glass, then, of the waters of Bonnes should be drank at first; this should be increased gradually to three or four glasses, drank pure, or dashed with milk, or oatmeal gruel. The same remark is applicable to all mineral waters which are very exciting, particularly to those of Canterets, of Barèges, of Bagnères de Luchon, of Enghien, of Mont d'Or, &c. Many practising physicians, forgetting the energy of these waters, advise patients whom they send to them to drink every morning at first four glasses, and to increase the quantity by degrees to two litres, (4.2 pints.) This dose, which can be supported but by a small number of the drinkers, produces various symptoms which the inspector is obliged to combat, and which are as prejudicial to the health of the patient as to the reputation of the waters.

The following is a statistical table of the chronic diseases of the chest, treated at Mont d'Or in the year 1837:

| NAMES OF DISEASES. | *Number of each disease.* | *Number of patients cured.* | *Number of patients relieved.* | *Number of patients treated without success* | *Number of patients cured or relieved after their departure from the watering place.* |
|---|---|---|---|---|---|
| Chronic pulmonary catarrh, complicated. | 22 | 4 | 11 | 7 | 5 |
| Chronic pneumonia. | 9 | 1 | 5 | 3 | 3 |
| Passive pneumorrhagia. | 17 | 2 | 8 | 7 | 4 |
| Pulmonary phthisis, at different stages. | 44 | 0 | 25 | 19 | 8 |
| Asthma. | 15 | 2 | 5 | 8 | 5 |
| Chronic pharyngo-laryngitis. | 18 | 4 | 10 | 4 | 3 |
| Aphony. | 9 | 4 | 2 | 3 | 2 |
| Nervous palpitations. | 3 | 1 | 0 | 2 | 0 |

Section 3. *On Chronic Diseases of the Abdomen.*—We have seen that mineral waters are rarely suited for the treatment of chronic diseases of the head, and that they succeed sometimes in that of diseases of the chest; they are, on the contrary, of considerable efficacy in chronic affections of the abdominal viscera; thus we meet at watering places a great number of these diseases.

*Lesions of the Digestive Canal.*—Nothing is more common at watering places than diseases known under the names of gastritis, chronic enteritis, gastralgia, dyspepsia, flatulence, pyrosis, colics, diarrhœa, &c. These lesions owe their origin to different causes; if they are due to a scirrhous or cancerous degeneration of one of the digestive organs, all mineral treatment is injurious. If they succeed to a phlegmasia, or if they are of a nervous character, change of air, light diet, and especially the internal use of the waters of Plombieres, Neris, and Luxeuil, or of acidulated waters, (Porges, Contrexville, Chateldon, Bussang, &c.,) have the advantage of modifying the mucous membrane of the stomach without irritating it; if there exists atony of the digestive canal, recourse should be had to the waters of Vichy, to ferruginous and sulphur waters. When the difficulty of digestion is attributable to a bilious or mucous condition of the gastro-intestinal canal, the laxative waters of Niederbronn, of Balaruc, and Lassere, at Bagnères de Bigorre, should be prescribed. In all cases it is necessary to insist upon the use of mineral baths, which, by producing an excitement and exanthemata upon the skin, effect a revulsion, and direct the blood from the centre to the circumference.* This revulsion is particularly useful, inasmuch as many of these diseases are produced by a suppression of the transpiration or a retrocession of a morbid principle. This last complication calls for the use of warm baths and vapour baths. In general, lymphatic or nervous individuals, affected with gastric irritations, find sea baths beneficial, which, by imparting activity to the peripheral circulation, provokes a salutary reaction of the skin.

*Engorgement of the Abdominal Viscera.*—When the gastro-intestinal lesions, of which we have just been speaking, are neglected, or aggravated by bad treatment, *obstructions*, commonly so called, or engorgements of the lower abdominal viscera are produced; diseases which are developed, also, under the influence of marsh miasma, and of obstinate intermittent fevers. Mineral waters may remove these engorgements when they are recent, passive, and when they are occasioned by a congestion of venous blood, or by a simple hypertrophy of the liver or spleen, without other alteration of tissue; for they are powerless if the viscus is affected with a tuberculous, cancerous, or fibrous degeneration. The waters of Vichy are justly the most famed for the cure of abdominal engorgements; but their use is abused; they only succeed when the patients are of constitutions but slightly irritable, and when there out other alteration of tissue; for they are powexist no farther traces of inflammation in the diseased viscus. The chalybeate waters of Spa, Forges, Passy, &c., are suitable, also, under like circumstances. For nervous individuals, and those in whom visceral irritation is not completely removed, the waters of Plombieres, and the cold acidulated waters of Pouges, Chateldon, &c., are to be preferred,—if there exists a bilious or mucous state, the laxative waters of Balaruc, and Niederbronn, by modifying the secretions of the liver, pancreas, and kidneys, and by expelling the bilious and mucous matters from the alimentary tube, produce a very marked resolutive effect, by means of which the chronic engorgements of the abdomen diminish or disappear. The chalybeate and saline waters of Bourbon and Balaruc succeed in cases of engorgements of the liver and spleen when they are consecutive to intermittent fevers, and in these same fevers when they resist cinchona. Out of twenty-two patients with abdominal engorgements who came to Bourbonne in 1836, six were cured, fifteen relieved, and one treated without success. M. Therrin, from whom we quote these results, cites the case of an officer who was cured by the use, alone, of the waters of Bourbonne, of a quartern fever, which had resisted all febrifuges. In 1750, a military surgeon, Juvet, of the asylum of Bourbonne, published several observations, which tend to prove that the mineral waters of this place cure intermittent quartern fevers better than cinchona. Two intermittent fevers treated at Balaruc, in 1837, were cured. At Crausac, where the waters are chalybeate, out of seventeen chronic hepatites, eight were cured, six were relieved, and three treated without success; out of 52 intermittent fevers, thirty-one were cured, 12 relieved, and nine treated without success.

If the chronic hepatites or splenites are produced by the retrocession of a rheumatismal principle, or of a cutaneous affection, vapour baths are very useful. The inspecting physician of Bagnols, (Lozere,) M. Blauquet, cites the case of a clergyman, who, after the disappearance of a rheumatism of the head, had hypertrophy of the liver, with jaundice; this patient was on the point of going to Vichy, when, reflecting upon the antecedents, M. Blauquet advised him to use the bath, the douches, and the vapour baths of Bagnols, which triumphed completely over the affection. When the engorgement is of a scrofulous nature, recourse must be had to chalybeate and sulphur waters; sea baths are used successfully in atrophy of the mesentery, (tabes mesenterica.)

*Chronic Diseases of the Urinary Passages.*—Most mineral waters, either on account of the quantity and the nature of the salts which they

* I have seen adults and children reduced to an extreme degree of emaciation by chronic diarrhœas, and so feeble, that it was found necessary to carry them to the bath, return, as it were, by magic, to a state of vigorous health, alone by the use of the baths of Plombieres. (*Notice sur les eaux de Plombieres, par M. Guersant, page* 17.)

contain, or on account of the large quantities in which they are sometimes drank, stimulate the kidneys, and increase the secretion of urine. Almost all inspecting physicians recommend their mineral waters in cases of nephritic colics and gravel. The springs of Contrexville, Pouges, and Bussang, and generally all the cold, acidulous waters are reputed as being particularly efficacious in the removal of calculi. They, in truth, greatly increase the secretion of urine, and augment the contractile power of the bladder, and are very good for expelling gravel; but they become injurious, increase the sufferings of the patient, and provoke, sometimes, hæmaturia when the stone is too large to pass the neck of the bladder. These waters are only diuretic, that is to say, they simply give tone and energy to the urinary organs, from which results the expulsion of small stones; they do not contain enough bicarbonate of soda to disaggregate and dissolve the vesicle calculi. It is only the waters containing a large quantity of this salt, such as the waters of St. Nectaire, Vals, and especially of Vichy, that possess this dissolving property. It appears from several facts, published by M. Charles Petit, that patients, who had all the rational signs of stone in the bladder, passed, after a use of the waters of Vichy, some of the nuclei of calculi, which were manifestly corroded and worn down, and presenting successive strata perfectly distinct; others, fragments and scales of calculous matter; and that, during their treatment, these patients found their symptoms of stone in the bladder to disappear gradually, and not again appear after a lapse of several months. Most physicians grant that the waters of Vichy have a real efficacy in cases of gravel, but they doubt that they can dissolve stones of a certain volume. To put this matter beyond dispute, it is indispensable that experienced surgeons should determine, with all possible precision, the volume of the stone before the mineral treatment, in order that, after it, the diminution in volume may be obtained by a second vigorous exploration. Such experiments alone can carry conviction to the minds of all; and M. Petit has proposed the performance of them to all surgeons, who, we hope, will hasten to reply to his call. The Minister of Commerce sent to the Academy, on the 16th of October last, a letter from this honourable member of our fraternity, in which he demands the formation of a committee to verify what he has said with regard to the efficacy of the waters of Vichy for the removal of urinary calculi. A committee has been named, and is now occupied on the subject of experiments; the results of their investigations they will communicate to you in due time.

*Chronic Diseases of the Genital Organs.*—Many mineral springs are in high repute for the cure of sterility; they do not act, then, by a specific property, but by removing the cause of this infirmity.* Thus, when it is to be attributed to a feeble constitution, to a too abundant leucorrhœal flow, or to a want of excitability in the womb, sulphur and chalybeate† waters, and sea bathing, by strengthening the system, may render females fruitful; if, on the contrary, the sterility is due to a nervous cause, to an excess of general or local sensibility, the waters of Ussat, of St. Sauveur, Neris, Bains, Bourbon-Laucy,‡ and Plombieres,§ are to be preferred. But, unfortunately, the causes of sterility are too often as mysterious as those of generation.

General debility, which is the consequence of masturbation, or of the abuse of sexual pleasures, involuntary seminal discharges, chronic gonorrhœas, relaxation of the ligaments of the uterus, leucorrhœa, caused by a sedentary life, are cured or relieved by the active waters of Mont d'Or, Bareges, Bagnères de Luchon, Bourbonne, and Balaruc, and especially by sea bathing. These waters easily bring about the monthly discharge, and hasten it ordinarily by a few days, which is very naturally explained by the excitement which they impress upon the circulatory system. They are also very salutary in chlorosis, in amennorrhœa, and dismenorrhœa, accompanied by languor, bloating, spasms, &c., and in uterine hæmorrhages, due to atony of the matrix. But if the suppression of the menses, or its too abundant flow, is owing to a local plethora, or to an excess of sensibility of the uterus, recourse must be had to the mild waters of St. Sauveur, Ussat, Neris, Bains, Luxeuil, &c. These springs are the best for women, who, at the critical period, complain of different nervous symptoms, which a too strong stimulation would not fail to aggravate.

---

* M. Bourdon, guide aux eaux minerales.

† It is to the visit paid by Louis XIII, and Anne of Austria, to the springs of Forges, (Seine Inferieure,) that historians attribute the disappearance of the sterility of this princess, who afterwards became pregnant with Louis XIV.

‡ Catherine de Medicis, the wife of Henry II., owed her fruitfulness to the waters of Bourbon-Laucy. Her physician, Fernel, having advised the use of these waters internally, in baths, and in douches, she had, at the end of nine months, Francis II., and, afterwards, Charles IX., and Henry III., who all reigned successively. At each accouchment she gave, in gratitude, to her physician thirty thousand francs, which was a large sum at that period.

§ Corvisart sent the Empress Josephine to Plombieres, but without success, for it was impossible to revive the vitality of an organ which had ceased to perform its functions.

STATISTICAL TABLE.

| NAMES OF DISEASES. | Name of establishment. | Number of each disease. | Number of patients cured. | Number of patients relieved. | Number of patients treated without success. | Number of patients cured or relieved after leaving the watering place. |
|---|---|---|---|---|---|---|
| Leucorrhœa. | Mount d'Or. | 13 | 5 | 3 | 5 | 0 |
| Atonic menorrhagia. | Id. | 3 | 0 | 1 | 2 | 0 |
| Chronic metritis. | Id. | 8 | 3 | 3 | 2 | 2 |
| Chlorosis. | Id. | 8 | 1 | 2 | 5 | 0 |
| Blenorrhagia. | Id. | 6 | 2 | 3 | 1 | 2 |
| General debility, in consequence of masturbation. | Id. | 9 | 2 | 3 | 4 | 4 |
| Amenorrhœa. | Sea-baths of Boulogne. | 2 | 1 | 1 | 0 | 0 |
| Dysmenorrhœa. | Id. | 2 | 2 | 0 | 0 | 0 |
| Leucorrhœa. | Id. | 3 | 1 | 2 | 0 | 0 |
| Displacement of uterus. | Id. | 3 | 3 | 0 | 0 | 0 |
| Dysmenorrhœa, from excitability of uterus. | Bains. | 15 | 9 | 4 | 2 | 0 |
| Atonic leucorrhœa. | Rennes, (Aude.) | 92 | 13 | 40 | 39 | 13 |
| Atonic amenorrhœa. | Id. | 35 | 10 | 0 | 25 | 6 |
| Atonic amenorrhœa. | Crausac. | 11 | 3 | 6 | 2 | 0 |
| Symptoms accompanying the critical period. | Bains. | 18 | 0 | 7 | 11 | 10 |

[*To be continued.*]

